# Hyperspectral Imaging for Mapping of Total Nitrogen Spatial Distribution in Pepper Plant

**DOI:** 10.1371/journal.pone.0116205

**Published:** 2014-12-30

**Authors:** Ke-Qiang Yu, Yan-Ru Zhao, Xiao-Li Li, Yong-Ni Shao, Fei Liu, Yong He

**Affiliations:** 1 College of Biosystems Engineering and Food Science, Zhejiang University, Hangzhou, China; 2 Key Laboratory of Equipment and Informatization in Environment Controlled Agriculture, Ministry of Agriculture, Beijing, China; University of Western Sydney, Australia

## Abstract

Visible/near-infrared (Vis/NIR) hyperspectral imaging was employed to determine the spatial distribution of total nitrogen in pepper plant. Hyperspectral images of samples (leaves, stems, and roots of pepper plants) were acquired and their total nitrogen contents (TNCs) were measured using Dumas combustion method. Mean spectra of all samples were extracted from regions of interest (ROIs) in hyperspectral images. Random frog (RF) algorithm was implemented to select important wavelengths which carried effective information for predicting the TNCs in leaf, stem, root, and whole-plant (leaf-stem-root), respectively. Based on full spectra and the selected important wavelengths, the quantitative relationships between spectral data and the corresponding TNCs in organs (leaf, stem, and root) and whole-plant (leaf-stem-root) were separately developed using partial least-squares regression (PLSR). As a result, the PLSR model built by the important wavelengths for predicting TNCs in whole-plant (leaf-stem-root) offered a promising result of correlation coefficient (R) for prediction (R_P_ = 0.876) and root mean square error (RMSE) for prediction (RMSEP = 0.426%). Finally, the TNC of each pixel within ROI of the sample was estimated to generate the spatial distribution map of TNC in pepper plant. The achievements of the research indicated that hyperspectral imaging is promising and presents a powerful potential to determine nitrogen contents spatial distribution in pepper plant.

## Introduction

Pepper (*Capsicum annuum* L) is one of the popular international spices in cooking [1], [2]. At present, world production of pepper is about 19 million tons fresh fruit from 1.5 million ha [3]. Nitrogen (N) is an essential element of vegetative growth, flowering, and fruit bearing of pepper plants [4]–[8], as well as in all metabolic processes of cellular structure and genetic coding [9]. In order to strengthen and stabilize plant growth and yield production, farmers most of the case will over-apply N fertilizer to ensure enough N for crop requirements. The excessive N fertilizer could result in a series of problems, such as lodging, delayed maturity, diseases, and weed [10], [11]. Therefore, it is crucial to investigate the N status and obtain the real-time spatial distribution information of N content in crops at different growth stages, so as to improve field management efficiency and economic benefit from agricultural production, as well as contribute to sustainable agriculture [12], [13].

Conventionally, measurement of total nitrogen content (TNC) in crops based on chemical analysis is a destructive measurement, which is high-cost and time-consuming [14]. Researchers demonstrated that TNC in plant was directly related to the formation of chlorophyll [15], [16]. Therefore, chlorophyll content in leaves can theoretically be considered as an indicator of the N status [17], [18]. The soil and plant analyzer development (SPAD) reading, an indirect value representing chlorophyll content, is measured by the SPAD meter with a “point-based” way [19]. This method merely provides information on chlorophyll content in leaves at one point, which is not sufficient to obtain the accurate chlorophyll concentration of the whole leaf. Moreover, SPAD meter cannot provide the chlorophyll information of other parts of the plant (e.g. buds, stems). In recent years, spectroscopic technology, particularly visible and near-infrared (Vis/NIR) spectroscopy, has been used for detection of the agricultural and sideline products. For plants, several researchers [20]–[24] employed spectroscopy technique to study and diagnose the nutrient status, N contents, and yield of pepper. Unfortunately, spectroscopic techniques fail to provide information on spatial distribution of quality parameters, which is essential to the analysis of products’ features [25]. Remote sensing technique can provide chlorophyll information of a large number of plants in an area with satellite or airplane sensors scanning. Airborne visible/infrared imaging spectrometer (AVIRIS) in NASA was used to determine forest canopy lignin, nitrogen, and ecosystem [26]. However, this approach is not suitable for providing chlorophyll information about individual plants [27].

Hyperspectral imaging combines conventional spectroscopy with imaging techniques to acquire both spectral and spatial information simultaneously for detecting physical, chemical, and biological attributes of the samples [28]–[30]. Relying on this capability, hyperspectral imaging has witnessed tremendous growth and been widely applied in agriculture [31]. Examples of nitrogen analyzing in plant included: mapping of foliar nitrogen of mangroves and explanation of spatial nitrogen variation in relation to local environmental variables [32], determination of nitrogen (N), phosphorus (P), and potassium (K) content and visualization of chemical distribution in oilseed rape leaves [33], estimation of leaf nitrogen accumulation in wheat [34], diagnostics of N deficiency by virtue of chlorophyll content in cucumber leaf [19], assessment of the leaf N content in wheat [35], estimation of canopy N concentration in temperate forest [36]. A majority of studies have investigated the chemical components in certain organ tissue (e.g. leaves) of crops. However, few studies on TNCs spatial distribution of plants using hyperspectral imaging have been reported.

In the current study, hyperspectral imaging technique was employed to map the spatial distribution of total nitrogen in pepper plant. The specific objectives were as follows: (1) to acquire hyperspectral images and measure TNCs of samples (leaves, stems, and roots) using Dumas combustion method; (2) to extract the spectral data and employ Random frog (RF) to select important wavelengths; (3) to build multivariate calibration models for predicting TNCs in organs (leaf, stem, and root) and whole-plant (leaf-stem-root) by partial least-squares regression (PLSR) based on full spectra and the selected important wavelengths; (4) to apply the optimal PLSR model to predict TNC of each pixel in samples and generate spatial distribution of TNCs in whole pepper plant.

## Materials and Methods

### Samples preparation

A total of 40 pepper plants (*Capsicum frutescens* L. *conoides* (Mill.) Bailey) were taken for this research. According to the crop management of National Farmers Information Service (NAFIS) [37], pepper plants were planted in the same environmental conditions and field management in greenhouses at Zhejiang University, Hangzhou (120°09′E, 30°14′N), China.

**Figure 1:**
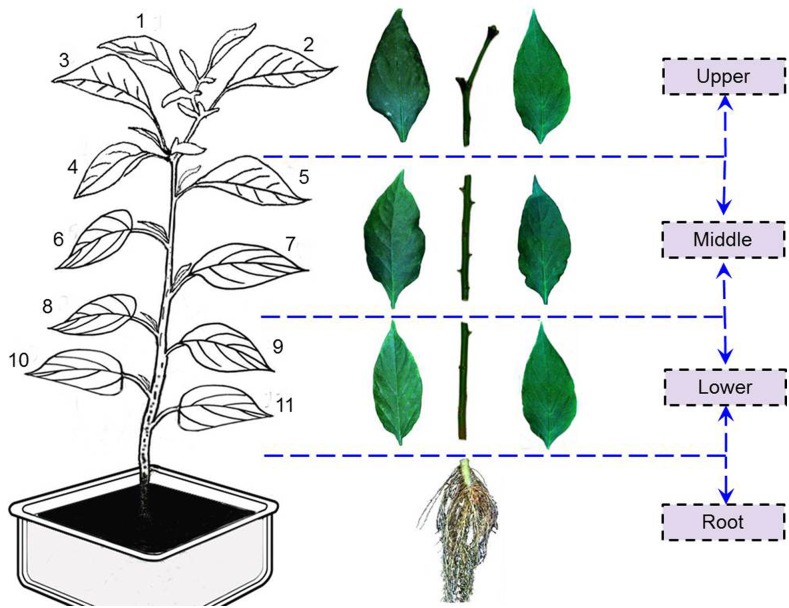
Positions of sampled leaf, stem, and root samples in one exemplary pepper plant.

After transplanting about 6 weeks, all pepper plants began to send forth flower buds during early blooming stage. According to the growth status of the pepper plants, several leaves (few yellow leaves in lower position and some small fresh leaves in upper position) were excluded, and then the remaining 11 true leaves with similar size were numbered from upper to lower (Fig. 1). In each pepper plant, 2 leaves (petioles were removed) were randomly sampled from the upper (1^st^–4^th^), middle (5^th^–8^th^), and lower (9^th^–11^th^) positions [38]. Each 2 leaves in the sampled position were viewed as a sample for subsequent data analysis, because the dry matter of one leaf failed to satisfy the demands of total nitrogen measurement. Meanwhile, the whole stem was divided into 3 parts according to positions (upper, middle, and lower) of petiole node. Lastly, root of each pepper plant was dug out from the soil and impurities were cleaned up. The position of sampled leaf, stem, and root is marked in Fig. 1. According to the preceding process, a total of 280 samples containing 120 leaf samples, 120 stems, and 40 roots were collected from 40 pepper plants. These samples were then used for hyperspectral data acquisition and TNCs measurement.

### Acquisition and calibration of hyperspectral images

Three-dimensional hyperspectral data of all samples were acquired by a laboratorial hyperspectral imaging device with the reflectance mode. The hyperspectral imaging device consists of: a mobile platform operated by a stepper motor (IRCP0076, Isuzu Optics Crop, Taiwan, China); an assembled illumination source with two 150-W quartz tungsten halogen lamps (Fiber-Lite DC950 Illuminator, Dolan Jenner Industries Inc., USA); an imaging spectrograph (ImSpectorV10E, Spectral Imaging Ltd., Finland) covering the spectral range of 380–1,030 nm with 512 wavebands; a CCD camera (C8484-05, Hamamatsu city, Japan) coupled with a zoom lens (OLES23, Specim, Spectral Imaging Ltd., Oulu, Finland); and a computer with the spectral imaging system V10E software (Isuzu Optics Corp, Taiwan, China), which is used to set and adjust the parameters of the device, including exposure time, motor speed, imaging acquisition, wavelength range, image correction. In all, the whole system (except the computer) was assembled in a dark chamber to minimize the effects of ambient light during the sample scanning.

As Yu et al. [39] described, the hyperspectral imaging device was calibrated before samples were scanned. Parameters of the device for image acquisition are summarized in Table 1. Subsequently, a hyperspectral image which was named “hypercube” with dimension of (*x*, *y*, *λ*) was generated with line scanning [39]. In detail, a hypercube contained *n*-pixels in *y*-direction (depending on the size of the sample), 672 pixels in *x*-direction, and 512 wavebands in *λ*-direction. Then, the raw hyperspectral images were calibrated with white and dark references using the following (1) and this process was implemented by the hyperspectral imaging analyzer software.
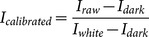
(1)where

**Table 1:** Parameters of hyperspectral imaging device for acquisition of images.

Parameters	Corresponding values
Spectral range	380–1,030 nm
Spectral resolution	2.8 nm
Moving speed of the mobile platform	2.1 mm/s
Spatial dimension of images	672×512 (spatial × spectral) pixels
Exposure time	0.008 s
The distance from lens to sample	295 mm

*I_calibrated_* is the calibrated hyperspectral image of the sample;

*I_raw_* is the raw hyperspectral image of the sample;

*I_white_* is the white reference image with 99% reflectance acquired from a white reference ceramic tile;

*I_dark_* is the dark reference image with 0% reflectance obtained by the camera lens thoroughly covered with its opaque cap.

### Measurement of TNCs using Dumas combustion method

The referenced method for TNC measurement was Dumas combustion using Nitrogen Analyzer Rapid N cube (Elementar Analysensysteme GmbH, Hanau, Germany). After hyperspectral images acquisition, all samples were dried completely to the constant weight [40]. Then, the samples were grinded by a Tissuelyser-48 (Jingxin Experimental Technology, Shanghai, China) operating in 70 Hz, 150 s. 50±1 mg dry matter of each sample was used to determine the TNC. After a series of processing (combustion, reduction, purification, detection, etc.), TNCs (%) of samples were obtained through the Rapid N software. All the measurements were carried out in a room at an approximate constant temperature of 25°C and a relative humidity of 20–30%.

### Hyperspectral data processing and analyzing

#### Hyperspectral data extraction

By implementing the mask function in the ENVI software, the target region of each sample was separated from hyperspectral image. The separated region was identified as the region of interest (ROI) of the corresponding sample. The spectra of each pixel in ROIs were then averaged and the average spectrum was considered as the spectrum of a sample. The spectra of other samples were obtained in the same way as mentioned above. Because of the low signal-to-noise ratio (SNR), the reflectance in two regions (380–420 nm and 1,000–1,030 nm) was rather low. Therefore, hyperspectral data were resized to the spectral range of 420–1,000 nm with 460 wavebands.

### Variable selection

Selection of important wavelength is of critical significance for removing the redundant information from high-dimensional data and optimizing calibration models for producing reliable results [41], [42]. Thus, identification of important variables carrying the most valuable and authentic information is a challenging task in the current hyperspectral data analysis [43].

In the present study, Random frog (RF) methodology was carried out to select important wavelengths. RF is a novel and efficient technique for variable selection, which borrows the framework of reversible jump Markov Chain Monte Carlo (RJMCMC) methods [44], [45]. It is employed to perform feature extraction for selecting a series of variables which describe the correlation between the predictor variables and the response variables [46]. In interior of RF algorithm, partial least squares regression (PLSR) is viewed as a modeling method. ***X*** (*n* × *p*) stands for the spectral matrix consisting of *n* samples in rows and *p* variables in columns. And ***Y*** (*n*×1) denotes the property of interest.

**Figure 2:**
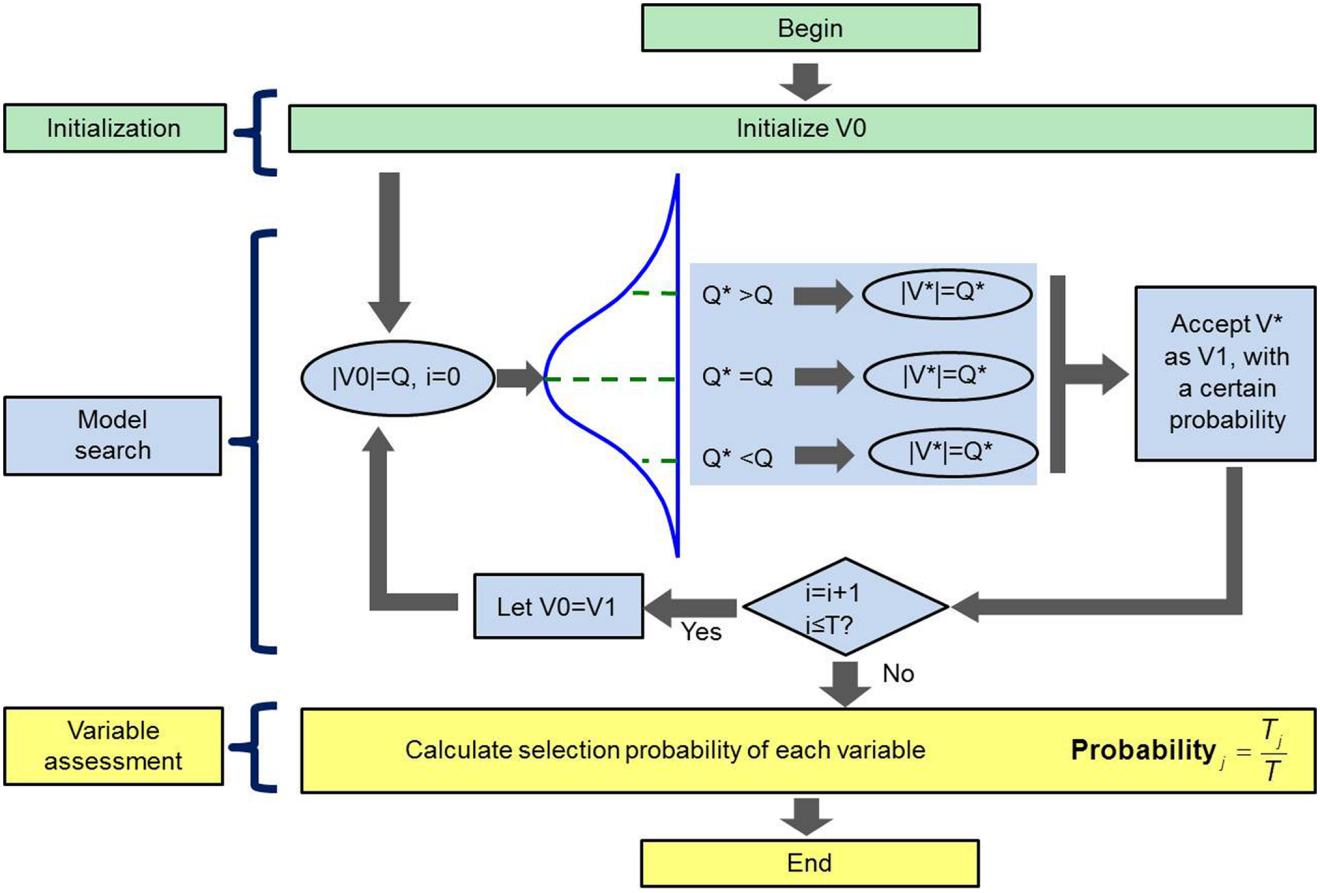
The flowchart of the Random frog algorithm from Li et al. [45].

Before running RF algorithm, five parameters (T, Q, *θ*, *ω*, and *η*, details were listed in Table 2) should be assigned with proper values. As shown in Fig. 2, random frog works in three steps [47]:

**Table 2:** Parameters for running random frog.

Parameters	Function	Value
T	number of iterations	10,000
Q	number of variables in the initialized variable set	50
*θ*	control variance of a normal distribution	0.3
*ω*	a coefficient explained in step 2	3
*η*	represent the upper bound of the probability	0.1

1. A variable subset V_0_ consisting of Q variables is initialized randomly;
2. A candidate variable subset V* is proposed based on Q* variables, accept V* with a certain probability as V_1_ and replace V_0_ using V_1_;
3. Selection probability of each variable after N iterations is computed. The details of RF algorithm could be found in literature [44], [45].

As Monte Carlo strategy is embedded in RF algorithm, the selection probability of variables is unable to be reproduced exactly [44], [45]. Generally, RF is implemented a couple of times (depending on the data) to minimize the influence of this random factor [44], [45].

### Multivariate data analyses

Partial least-squares regression (PLSR), one of the reliable analytical tools for modeling [48]–[51], has a wide application in multivariable data analysis and regression. It possesses an advantage to solve the situation when the number of variables is larger than that of samples, and when there is colinearity among variables [52]. The PLSR model employs several latent variables (LVs) instead of real variables and develops the calibration model applying leave-one-out cross-validation (LOOCV) method [53], [54]. The LOOCV method is applied to validate the performance and evaluate over-fitting of the calibration models. In this study, the quantitative relationships between spectral reflectance values and TNCs of samples were established by employing PLSR.

### Evaluation of models

The performances of models were evaluated by correlation coefficient (R) and root mean square error (RMSE) across the calibration (R_C_, RMSEC), cross-validation (R_CV_, RMSECV), and prediction (R_P_, RMSEP) sets of samples. Generally, an optimal model should provide high R values, low RMSE values, and a small difference between calibration and prediction [40].

### Chemical imaging

Chemical imaging is a technique for building visual color images to display the spatial distribution of chemical components in heterogeneity [55]. The chemical value of each pixel can be predicted by inputting its corresponding spectral reflectance values into the established quantitative model [19]. Spatial distribution of chemical components could be generated with the established quantitative model combined with image processing. To observe the variance of TNCs in whole pepper plant, distribution maps of TNCs were required. In particular, an optimal model built using the mean spectra of important wavelengths was applied to predict the TNC in each pixel. Subsequently, the spatial position of each pixel along with its TNC was used to form the spatial distribution maps [52].

### Software tools

The measured TNCs statistics and analysis of variance (ANOVA) was finished using Microsoft Office Excel 2010 and IBM SPSS Statistics (version 20.0, IBM Corporation, Armonk, New York, USA). Hyperspectral data was extracted with ENVI software (Version 4.6, ITT Visual Information Solutions, Boulder, USA). The processes of spectral data analyses were carried out by “The Unscrambler X10.1” (CAMO PROCESS AS, Oslo, Norway). In addition, Random frog (version 2.0) toolbox and the developed program for building TNC spatial distribution maps were worked in MATLAB 7.8 (R2009a) software (The MathWorks, Inc., Natick, MA, USA). OriginPro 8.0 SR0 (Origin Lab Corporation, Northampton, MA, USA) software was employed to design the spectral curves. All processes were run on a PC (CPU: Intel Core i3-3220 @3.30 GHz, RAM: 4.00 GB) operating with Windows 7.

## Results and Discussion

### Statistics of the TNCs measured by Dumas combustion (TNCs-DC)

The statistical results of TNCs in pepper plant samples measured by Dumas combustion are presented in Table 3. The results were similar to previous study of Zhang et al. [33], Nelson et al. [56], and Stevenson [57]. The mean TNCs-DC in leaves (3.295%), roots (1.186%), and stems (0.992%) displayed a descending tendency. Meanwhile, the TNCs-DC in groups of leaf and stem conveyed an ascending trend in lower/middle/upper positions, ranging from 2.703% to 3.129%, then to 4.053% in leaves, and from 0.736% to 0.849%, then to 1.390% in stems. Also in Table 3, results of analysis of variance (ANOVA) analyses of the TNCs-DC in different samples revealed an apparent difference among leaf/stem/root groups, as well as the leaf and stem groups in upper/middle/lower positions.

**Table 3:** Statistic results of all samples’ TNCs-DC.

Samples	Positions	N.[1]	Mean (%) ± S. D.[2]
Leaf	Upper	40	4.053±0.498^a^
	Middle	40	3.129±0.321^b^
	Lower	40	2.703±0.325^c^
Stem	Upper	40	1.390±0.366^a^
	Middle	40	0.849±0.175^b^
	Lower	40	0.736±0.102^c^
Leaf	All	120	3.295±0.686^a^
Stem	All	120	0.992±0.374^c^
Root	All	40	1.186±0.143^b^
Leaf-root-stem	All	280	2.007±1.231

Note: Different letters (a, b, c) in the same column indicate statistical significance at the 5% level by Tamhane’s T2 test.

[1] N.: Number of samples;

[2] S. D.: Standard deviation of the group.

One-way analysis of variance (ANOVA) was used to generate these results, which were obtained using IBM SPSS Statistics (Version 20.0, IBM Corporation, Armonk, New York, USA). The results of TNCs-DC exhibited significant differences between groups (leaf, stem, and root) and within groups (leaves/stems in different positions).

In this research, the SPXY (sample set partitioning based on joint *x*–*y* distances) method proposed by Galvao et al. [58] was implemented to divide all samples into calibration sets with 210 samples (90 leaves, 90 stems, and 30 roots) and prediction sets with 70 samples (30 leaves, 30 stems, and 10 roots) for subsequent hyperspectral TNC analyses. Table 4 shows a summary of statistical analysis on the TNCs-DC of all samples in calibration and prediction sets. There was an evident variation in TNCs-DC in the calibration set, ranging from 2.264% to 4.871% for leaf, 0.847% to 1.470% for root, 0.556% to 2.135% for stem, and 0.556% to 4.871% for whole-plant samples. Samples with a wide compositional distribution of the TNCs-DC were collected, which was of great importance to build stable calibration models.

**Table 4:** Summary of statistical analyses of TNCs-DC in calibration and prediction sets.

Samples	Calibration set				Prediction set			
	N.[1]	Max.[3] (%)	Min.[4] (%)	Mean (%) ± S.D.[2]	N.[1]	Max.[3] (%)	Min.[4] (%)	Mean (%) ± S.D.[2]
Leaf	90	4.871	2.264	3.357±0.701	30	4.731	2.398	3.108±0.612
Root	30	1.470	0.847	1.183±0.156	10	1.301	1.035	1.194±0.103
Stem	90	2.135	0.556	1.040±0.403	30	1.636	0.599	0.849±0.218
All	210	4.871	0.556	2.114±1.292	70	4.731	0.599	1.927±1.218

Note:

[1] N.: Number of samples;

[2] S. D.: Standard deviation of the group;

[3] Max.: Maximum;

[4] Min.: Minimum;

Calibrations set with wide variation range of TNCs-DC could benefit for building robust models. Cross-validation set had the same results with the calibration set, which were not motioned in this table. In this study, leave-one-out cross-validation (one sample randomly chosen from calibration set was retained at a time and the rest of samples in calibration set were used to build the model) was used to verify the reproducibility and robustness of models.

### Overview of spectral features

**Figure 3:**
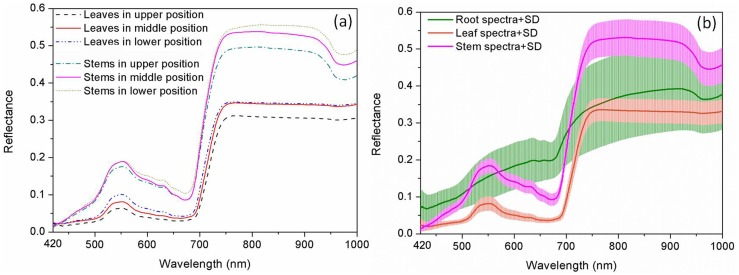
Spectral curves of all pepper plant samples covering the range of 420–1,000 nm. (a) mean spectral reflectance curves of all leaves and stems in upper, middle, and lower positions; (b) mean spectral reflectance and standard deviation (SD) of leaves, stems, and roots across all samples.

Fig. 3 demonstrates the mean spectral reflectance curves of samples covering the range of 420–1,000 nm. In Fig. 3(a), there were similar profiles of curves of leaves and stems in upper/middle/lower positions, however, the discrepancy of two groups only appeared on the different spectral reflectance values. Moreover, spectral reflectance of leaves and stems in lower/middle/upper positions displayed an ascending gradient. In detail, molecular vibration around 550 nm is caused by chlorophyll, which related to N concentrations [20], [32]. Small valley of the curves around 960 nm is attributed to the O-H second overtone, which is related to water in plants [19], [39], [41], [55], [59]. The region of 680–750 nm with high reflectance values generally refers to the “red edge” [60].

The mean spectral reflectance and standard deviation (SD) of leaf/stem/root groups are illustrated in Fig. 3(b). The mean reflectance and SD of the stem was consistently higher than that of the leaf across the whole tested range. There were several intersections of means for each three groups (roots, stems, and leaves), which was attributed to their different tissue structures. The spectra of organs (leaf, stem, and root) resulted from a complex combination of scattering processes and overlapping absorptions arising from water and biochemical components [61]. In addition, the trend of the root curve was relatively flat and different from those of leaf and stem, both of which were similar to the study reported by San Juan Martínez [21]. Actually, some soil particles adhered to the surface of root, resulting in spectrum similar to the soil where the plant grew [21], [62].

### Selection of important wavelengths using Random frog algorithm

**Figure 4:**
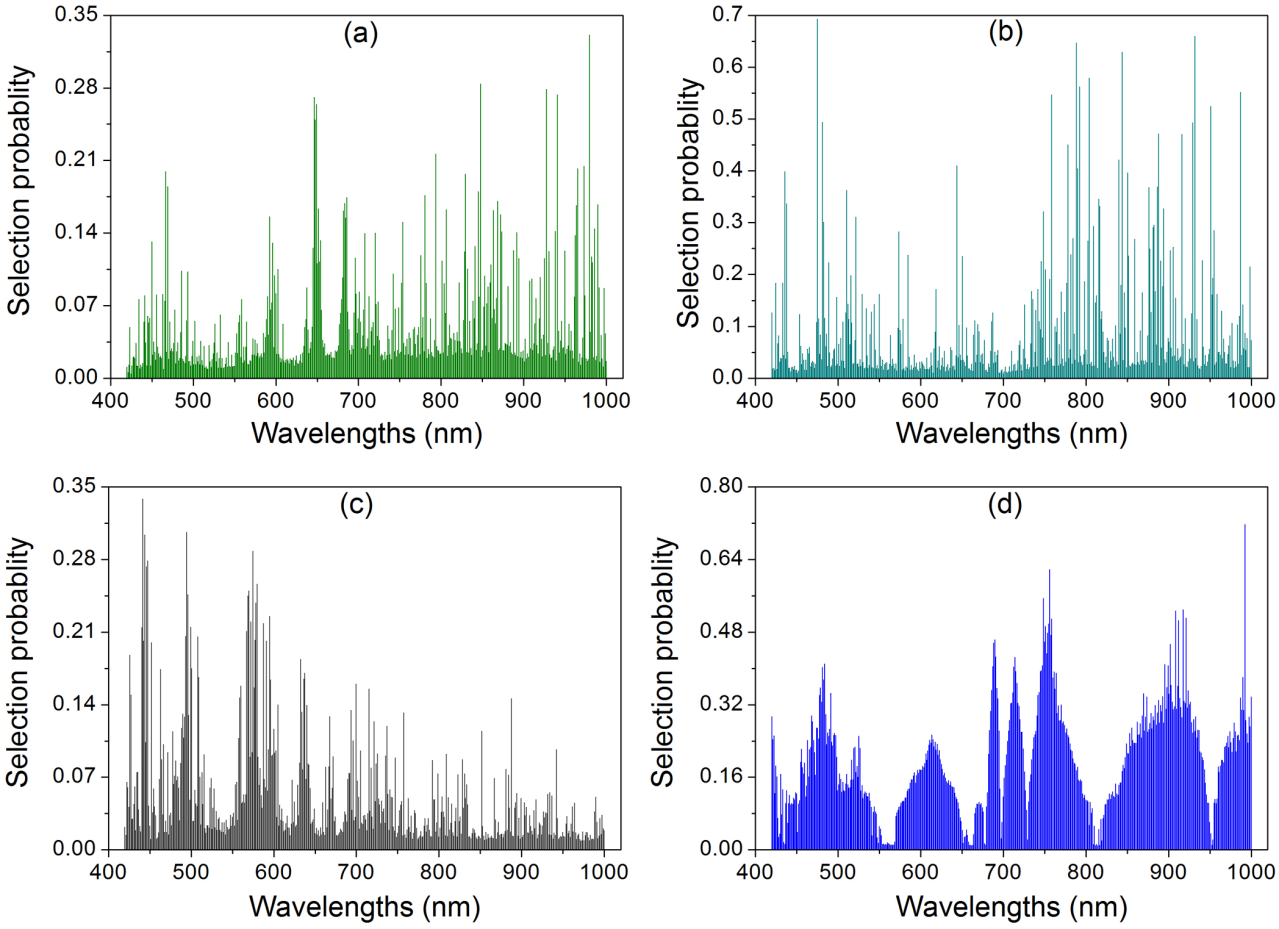
Selection probability (SP) of each wavelength averaged over 50 runs of Random frog for TNCs-HSI prediction of (a) leaves, (b) stems, (c) roots, and (d) whole-plant samples (leaf-stem-root).

In this research, RF was executed 50 times and the average value over these 50 runs was taken as the criterion for estimating the importance of each variable. The selection probability (SP) of wavelengths is shown in Fig. 4. From these SP curves, it could be found that a small number of wavelengths exhibited an extremely high SP, whereas the SP of most wavelengths was relatively low. This indicated that the majority of wavelengths provided a weak relevance to the TNCs predicted by hyperspectral imaging (TNCs-HSI). All the variables were ranked in descending order, according to the SP. To investigate the influence of the number of wavelengths in the model, as well as to seek an optimal number of variables [45], different wavelengths for benefiting the TNCs-HSI in organs (leaves, stems, and roots) and the whole-plant (leaf-stem-root) are collected in Table 5.

**Table 5:** Important wavelengths for predicting TNCs in leaves, stems, roots, and whole-plant (leaf-stem-root) based on Random frog (RF).

Samples	Important wavelength (nm)
Leaves	980, 848, 928, 941, 647, 649, 648, 794, 973, 965
Stems	475, 932, 788, 844, 804, 792, 987, 759, 951
Roots	445, 495, 444, 575, 448, 446, 580
Whole-plant	992, 756, 749, 918, 909, 921, 759, 912

The selected important wavelengths of leaves were mainly scattered in three regions (around 650, 790, and 970 nm), in agreement with previous researches [22], [23], [33], [31]. The important wavelengths for TNCs-HSI in stems were principally clustered in 790 and 980 nm. However, the important wavelengths of root were largely distributed around the regions of 445 and 580 nm, which were similarly related to the wavelengths characteristic for soil [21], [63]. For whole-plant (leaf-stem-root), the selected important wavelengths at 756, 749, and 759 nm were assigned to the third overtones of N-H stretching around 785 nm [31], [64], [65]. The selected important wavelength at 992 nm was close to the second overtones O-H stretching near 970 nm [39], and the remaining selected important wavelengths at 918, 909, 921, and 912 nm were ascribed to the third overtones of free monomer C-H stretching (934 nm) in carboxylic acid [31].

### Establishment of the PLSR models

The PLSR predictive models of the TNCs-HSI in organs (leaf, stem, and root) and whole-plant (leaf-stem-root) were separately established based on the full spectra and the selected important wavelengths. The results of these models are enumerated in Table 6. Organ prediction methods provided more accurate TNC estimates, compared with the whole-plant model. Meanwhile, PLSR models based on both full spectra and the selected important wavelengths showed indeed only minute changes between the calibration and the respective cross-validation values. However, organ prediction methods needed three models to accrue TNCs-HSI in different organs, which enhanced the complexity of determining the TNCs-HSI of whole-plant. Furthermore, the pepper plant should be taken as a whole experimental subject. Hence, the whole-plant models with more samples (the sum of roots, stems, and leaves) were introduced to obtain TNCs-HSI in pepper plant and obtain good performance.

**Table 6:** Results of PLSR models for TNCs-HSI analysis based on full spectra (F-PLSR) and the selected important wavelengths (RF-PLSR).

Samples	Models	N.[1]	LVs	Calibration		Cross-Validation		Prediction	
				R_C_	RMSEC (%)	R_CV_	RMSECV (%)	R_P_	RMSEP (%)
Leaf	F-PLSR[5]	460	17	0.974	0.156	0.924	0.269	0.934	0.223
	RF-PLSR[6]	10	3	0.817	0.401	0.798	0.420	0.828	0.356
Stem	F-PLSR	460	16	0.987	0.063	0.917	0.168	0.930	0.084
	RF-PLSR	9	5	0.820	0.229	0.773	0.254	0.724	0.157
Root	F-PLSR	460	11	0.992	0.020	0.931	0.064	0.915	0.045
	RF-PLSR	7	7	0.773	0.097	0.592	0.130	0.797	0.068
Whole-plant	F-PLSR	460	6	0.904	0.388	0.893	0.409	0.908	0.351
	RF-PLSR	8	4	0.878	0.451	0.874	0.468	0.876	0.426

Note:

[1] N.: Number of wavelengths used for analysis;

[5] F-PLSR models meant the PLSR models established by using full spectra;

[6] RF-PLSR models represented the PLSR models built by important wavelengths selected by RF algorithm. LVs, R_C_, RMSEC, R_CV_, RMSECV, R_P_, and RMSEP could be found in the text.

From the results of whole-plant models (Table 6), the PLSR model based on full spectra (F-PLSR) with 6 latent variables (LVs) provided relatively robust results to benefit the TNCs-HIS, compared with PLSR model built using important wavelengths selected by RF (RF-PLSR). When switching from F-PLSR model (406 variables) to RF-PLSR model (8 variables), 98.26% of variables were eliminated. Meanwhile, the correlation coefficients of calibration, cross-validation, and prediction (R_C_, R_CV_, and R_P_) only showed a slight reduction of 0.026, 0.019, and 0.032, while their respective errors RMSEC, RMSECV, and RMSEP exhibited small changes of 0.063, 0.059, and 0.075. It meant that the predictive power of the RF-PLSR model would slightly drop for the whole-plant TNCs-HSI prediction in comparison with the F-PLSR model. However, the F-PLSR model with 460 wavelengths (varieties) would bring about a time-consuming data process [51], [53], and failed to offer a simple and effective linear for predicting the TNCs-HSI. In addition, several models were less over-fitted, which might be caused by the improper values of LVs. The above results revealed that the RF-PLSR model of whole-plant proved best-suited for gaining the TNCs-HSI in pepper plants.

The promising quantitative relationship between the spectral reflectance values and the TNCs-DC of samples was established through the RF-PLSR model. A multi-linear function for the TNCs-HSI prediction of the whole-plant (leaf-stem-root) was obtained:
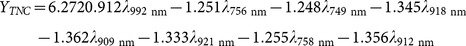
(2)where

λ_i nm_ is the spectral reflectance value at the wavelength of i nm;

*Y_TNC_* is the TNCs-HSI estimate of whole pepper plant.

### Spatial distribution maps of TNCs in pepper plant

**Figure 5:**
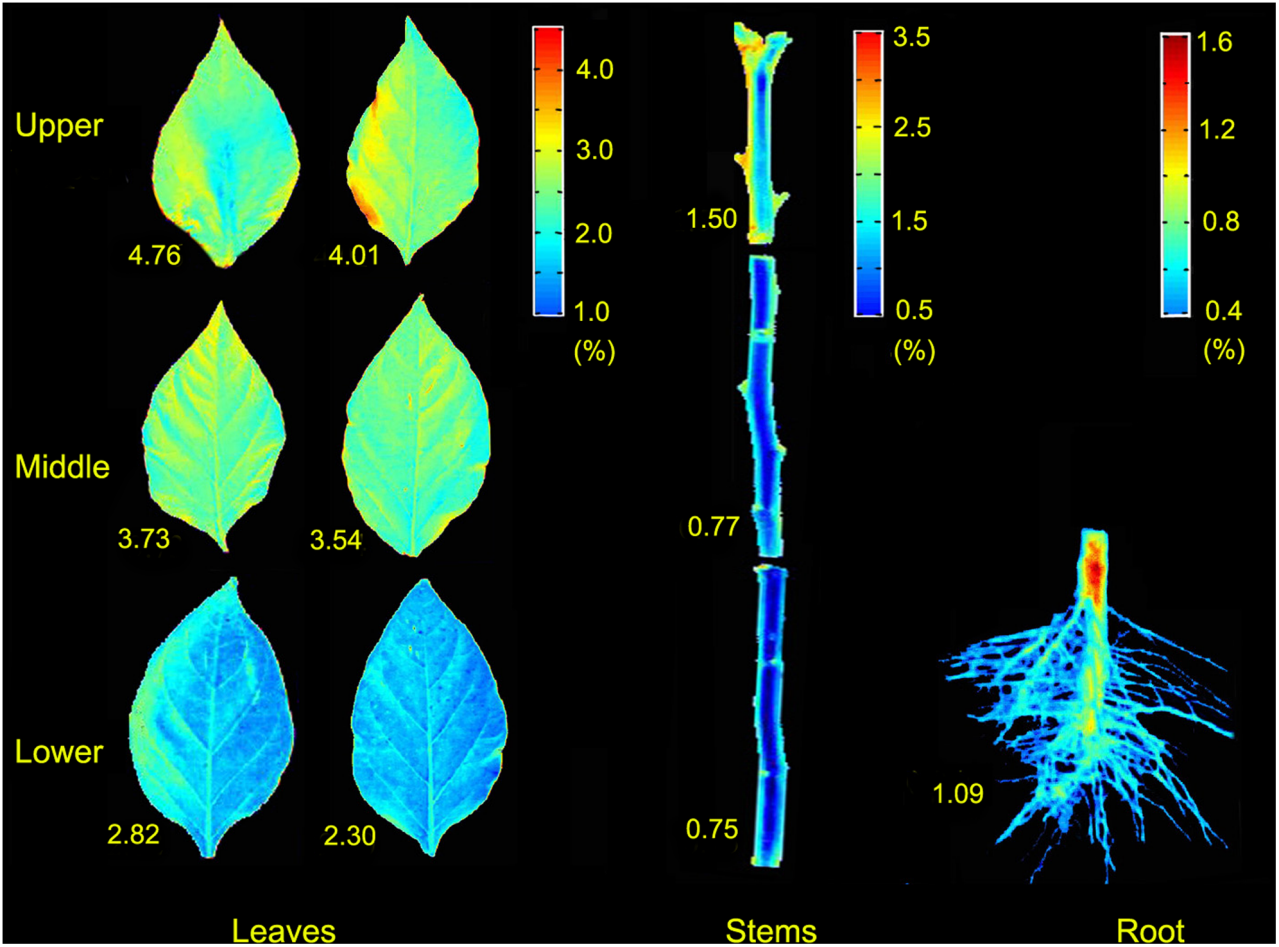
Spatial distribution maps of TNCs-HSI in samples of an exemplary tested pepper plant included six leaves, three stems, and one root, respectively. TNCs-HSI of samples in hyperspectral images were computed based on linear function (2) and TNCs-HSI distribution was achieved in MATLAB software. The numbers accompanying each sample map denote the respective TNC-DC value.

The linear function (2) obtained from the RF-PLSR model was employed to predict the TNC of each pixel within the leaf, stem, and root images. Pixels with similar spectral patterns in raw hyperspectral images would produce similar predicted values of the TNCs-HSI, and then would appear in similar colors in the resulting chemical images [55]. As a result, the spatial distribution maps of the TNCs-HSI in 10 samples (6 leaves and 3 stems in upper, middle, and lower positions and a root) of pepper plant were generated in Fig. 5. The TNCs-DC covered a broad range from 0.75% (stem in lower) to 4.76% (leaf in upper). Pixels providing similar spectral information in original hyperspectral images would result in similar results of the TNCs-HSI [51], [55], thus producing similar colors in the resultant chemical images. Indeed some samples (stems in middle/lower and root) with the relatively low TNCs-DC might show similar color, which was hard to distinguish based on the pixels of high or low TNCs. Hence, in the predictive map, three color scales described the ranges of the TNCs-HSI in each spot of the leaves, stems, and root samples, respectively. Compared with the images of original samples, the difference of the TNCs-HSI coloring within a sample could be easily identified by the naked eyes.

As shown in Fig. 5, three linear color scales in different colors from red to blue represented the different TNCs-HSI from high to low, respectively. The 10 resulting images of samples revealed the changing spatial tendency of the TNCs-HSI. Nitrogen status showed a decreasing trend from upper to lower positions in leaves and stems, which was in alignment with the measured nitrogen variation shown in Table 3 and the respective TNC-DC values of those samples. This phenomenon could be explained as follows: N is a removable and active element and exists in the form of organic matter in a living plant. It can be reabsorbed from older leaves to young leaves [19]. Meanwhile, N and other nutrients in plant are transported from roots to growth center (fresh leaves) with the xylem sap in stems under the transpiration pull [66], [67], resulting in the high TNCs in fresh leaves. In addition, TNC distribution is related to seasons, plant age, and photon flux density of leaves in different positions [68].

According to plant physiology [69]–[72], N element is absorbed on the root surface in the form of ammonium (NH4^+^) or nitrate (NO3^−^) by the movement of water in the soil [73]. Then, N ions enter the conduit of xylem in the root tissues through the apoplast pathway. The sap ascending in the xylem of nitrogen-fixing legumes carries nitrogen compounds originating from inorganic soil nitrogen (mainly NO3^−^ absorbed by the roots). Early growth of the leaf depends largely on phloem for intake of nitrogen and other nutrients that transported with xylem. At last, nutriments are transported from roots to various organs and tissues through the xylem sap under the physiological activities.

It is worth noticing that the maps of the upper and middle leaves displayed a relatively even color within the mesophyll part of individual images. In the images of the lower leaves (Fig. 5), pixels in the midrib part with light blue implied the relatively high TNCs-HSI. For stems, petiole nodes with orange showed the relatively low TNCs-HSI. In the map of root, hair parts colored blue revealed the extremely low TNCs-HSI, as well as in stems and leaves.

Furthermore, colors in the contour of stem samples were distinctly different in other regions, which might be caused by its tridimensional structure. Samples with the planar or tridimensional structure might result in different spectral characteristics [74]. This issue would be deliberated in further work.

### Conclusions

In this research, RF method was employed to select eight important wavelengths (992, 756, 749, 918, 909, 921, 758, and 912 nm) for successful prediction of whole-plant (leaf-stem-root) total nitrogen contents. After that, PLSR was employed to build the quantitative relationship between the spectral reflectance and TNCs-DC of samples based on full spectra and important wavelengths. The RF-PLSR model of whole-plant with results of R_P_ = 0.876 and RMSEP = 0.426% was considered the optimal model for the TNCs-HSI prediction in pepper plants. Lastly, the TNCs-HSI of all pixels in samples were calculated by applying the optimal PLSR model. Meanwhile, spatial distribution maps of the TNCs-HSI in pepper plant samples were constructed by using a developed image processing procedure. It could be inferred that the differences in TNCs within pepper plant could provide important information for monitoring nutrient distribution.

In further research, more varieties of pepper plant with different geographical locations, ages, time, and broader chemical values should be taken into account for developing more adequate and accurate TNC prediction models, which could provide a theoretical guidance for crop nutrient diagnosis and field management.
