# Correction: Hyperspectral Imaging for Mapping of Total Nitrogen Spatial Distribution in Pepper Plant

**DOI:** 10.1371/journal.pone.0134071

**Published:** 2015-07-24

**Authors:** The PLOS ONE Staff

In the Results and Discussion, there is an error in the second equation in the “Establishment of the PLSR models” subsection. The publisher apologizes for the error. Please view the complete, correct equation here:

$$Y_{TNC}=\;6.272–0.912\lambda_{992\;\text{nm}}-1.251\lambda_{756\;\text{nm}}-1.248\lambda_{749\;\text{nm}}-1.345\lambda_{918\;\text{nm}}-1.362\lambda_{909\;\text{nm}}-1.333\lambda_{921\;\text{nm}}-1.255\lambda_{758\;\text{nm}}-1.356\lambda_{912\;\text{nm}}$$

Where λ_i nm_ is the spectral reflectance value at the wavelength of i nm; *Y*_*TNC*_ is the TNCs-HSI estimate of whole pepper plant.
